# Intraoperative Intravenous Methadone and Postoperative Opioid Requirements in Adult Patients With Burns

**DOI:** 10.1093/jbcr/iraf209

**Published:** 2025-11-05

**Authors:** Christopher R LaChapelle, Aditee Ambardekar, Jenny Ringqvist, Aiden Berry, Paul Nakonezny, Anthony Dao, Sarah Rebstock

**Affiliations:** Department of Surgery, University of Utah Health, Salt Lake City, UT, United States; Department of Anesthesiology and Pain Management, University of Texas Southwestern, Dallas, TX, United States; Department of Anesthesiology and Pain Management, University of California Davis, Sacramento, CA, United States; Department of Anesthesiology and Pain Management, University of Texas Southwestern, Dallas, TX, United States; Department of Anesthesiology and Pain Management, University of Texas Southwestern, Dallas, TX, United States; Department of Anesthesiology and Pain Management, University of Texas Southwestern, Dallas, TX, United States; Department of Anesthesiology and Pain Management, University of Texas Southwestern, Dallas, TX, United States

**Keywords:** methadone, burn, pain, adult

## Abstract

Postoperative pain management is a significant challenge in patients undergoing burn excision. Pharmacologic pain management strategies include both opioid and non-opioid medications. Given the national overuse of opioids and the associated negative effects, it is prudent and essential to find ways to manage pain with fewer or no opioids. We hypothesize that intraoperative administration of intravenous methadone reduces total morphine milligram equivalents per weight used in the 36 h following surgery. This is a retrospective, single-center cohort study of adult burn patients who underwent a first excision of full thickness burn between January 2019 and January 2021. One group received intraoperative intravenous methadone while the non-exposure group did not. The primary outcome was total morphine milligram equivalents per weight utilized in the 36 h following surgery. Secondary outcomes included average pain scores in the post-anesthesia care unit and for 36 h postoperatively, as well as discharge opioid prescriptions. The methadone group contained 104 subjects, and the non-exposure group contained 119 subjects. Poisson regression, with adjustment for covariates, showed that the methadone group required fewer 36-h postoperative opioids (IRR = 0.89, *P* = .447) and were discharged with fewer opioid prescriptions (IRR = 0.86, *P* = .363) independent of the age and %TBSA differences. Post-anesthesia care unit pain scores were lower in the methadone group (IRR = 0.91, *P* = .350), as were 36-h postoperative pain scores (IRR = 0.92, *P* = .310). These trends toward improved pain control and reduced opioid requirements in patients receiving intraoperative, intravenous methadone did not reach statistical significance. Prospective, adequately powered randomized studies are needed to advance these findings.

## INTRODUCTION

Postoperative pain control remains one of the most significant clinical challenges in the management of patients undergoing surgical excision of burn wounds. Failure to appropriately manage a patient’s acute burn pain can lead to delayed discharge and higher rates of psychological sequelae.^1–3^ It is recognized by most burn clinicians that opioid pain medications are necessary to appropriately manage patients with significant thermal injuries, but their use is not free of risk for complications.^4–6^ Recent guidelines released by the American Burn Association (ABA) call for a multimodal approach in managing acute burn pain, including both pharmacologic and non-pharmacologic strategies.^4^ These strategies may reduce the use of opioids and reduce the effects of chronic pain and opioid use disorder which may develop, but do not eliminate the need for opioids.^4^

Short-acting opiate medications are frequently utilized intra- and post-operatively; however, due to their pharmacodynamics and pharmacokinetics, rapid swings in blood concentrations require frequent redosing and can lead to complications, including respiratory depression, cardiovascular instability, drowsiness, nausea, and vomiting.^6^^,^^7^ With repeated administration of opiates, patients may also develop tolerance and hyperalgesia.^4^^,^^8^^,^^9^

An alternative option for managing acute and chronic burn pain is the long-acting opioid, methadone. Methadone is an attractive medication for several reasons. When given parenterally, it is rapid in onset and slow to clear from the bloodstream.^7^ This results in a steadier concentration of the drug over longer time. The benefits of this stem from activity on traditional mu receptors and antagonism at the N-methyl-D-aspartate (NMDA) receptor, which may mitigate opioid tolerance, hyperalgesia, and chronic pain syndromes post-surgery.^10^^,^^11^ It also has been shown to inhibit reuptake of serotonin and norepinephrine in the CNS, which may provide benefit in the postoperative period through mood modulation and descending 5HT-3 inhibition.^12–14^ Methadone therefore seems to be an ideal opioid in the management of surgical and post-surgical burn pain.

Previous studies have supported the use of intraoperative methadone in spine, cardiac, obstetric/gynecologic, and general surgical populations to decrease the use of other opioid medications; however, meta-analyses of mixed populations have reported varied results.^15–17^ The literature for the use of methadone in the burn population is limited to small case series and its use in mostly intubated patients.^7^ Previous research in the pediatric burn population showed a reduction in postoperative opioid use with administration of intraoperative methadone.^18^

The aim of this study was to compare differences in the required opioid medications for 36 h postoperatively in adult burn patients undergoing their first burn excision who received a single dose of intravenous methadone intraoperatively with those who did not. The secondary aims of this study are to evaluate differences between groups for pain scores and opioids prescribed at discharge. We hypothesized that intraoperative administration of intravenous methadone would reduce total morphine milligram equivalents per weight used in the 36 h following surgery and at discharge.

## METHODS

### Procedures and measures

This is a single center retrospective cohort study at a Level 1 trauma center with an ABA verified burn center. Institutional Review Board approval was obtained prior to initiating this study.

All burn patients ≥18 years of age who underwent their first surgical excision of burn wounds with or without autografting were identified from the electronic medical record between January 2019 and December 2021. Patients who were intubated in the burn unit before or after surgery were excluded, as it was difficult to compare patient reported pain scores with nurse-derived pain scores. Pediatric patients were also excluded. Included patients were then divided into 2 groups: those who received intraoperative methadone (methadone group) and those who did not (non-exposure group).

All data were extracted from the electronic medical record by trained research staff. Demographic data were collected and included patient age, sex, height, weight, body mass index (BMI), and comorbidities. Total body surface area (TBSA) and presence/absence of donor site were recorded. The presence of a donor site was used as a surrogate for an autografting procedure, whereas the absence of a donor site was used as a surrogate for a temporary coverage procedure. Data on opioid and non-opioid pain medications and respective doses were recorded for 3 time periods/locations: intraoperative, post-anesthesia care unit (PACU) and for the total 36 h postoperatively. The total 36-h postoperative opioid consumption included both time in the PACU and time back in the burn center. All opioid medication doses were converted to intravenous milligrams of morphine equivalents (MME) using the Practical Pain Management Calculator and standardized between patients by weight in kilograms (MME/kg).^19^ Opioid prescriptions at discharge were also recorded. Hand-written or missing discharge prescriptions from the electronic medical record were identified in the prescription monitoring program state database.^20^

### Outcome variables

The primary outcome was MME/kg for 36 h postoperatively. The secondary outcomes were average pain scores measured in the PACU and for the 36 h postoperatively, as well as MME/kg prescribed at discharge.

### Statistical analysis

Demographic and clinical characteristics for the evaluable sample were described using the sample mean (SD) for continuous variables and the frequency (%) for categorical variables. To identify any differences between the characteristics of patients who received intraoperative methadone and those who did not, the 2-independent sample *t*-test, with the Satterthwaite method for unequal variances, and Fisher’s exact test (categorical variables) were used.

Poisson regression was used to model the mean of the discrete response variables of the total number of MME/kg administered in the 36 h following surgery and at discharge, as well as pain scores measured in the PACU and 36 h postoperatively. Separate Poisson regression models, with maximum likelihood estimation, were implemented and were used to account for the right skewness (and zero counts) of both outcomes; thus, providing efficient parameter estimates in evaluating the effect of methadone on the outcomes. Clinically relevant covariates of age, sex, BMI, %TBSA burned, donor site status total intraoperative MME/kg, intraoperative ketamine and postoperative acetaminophen, gabapentin, ibuprofen, and methocarbamol were included in each model to evaluate the independent effect of methadone on the outcomes. The Poisson models were checked for overdispersion. The incidence rate ratio (IRR) was reported, which was obtained by exponentiating the Poisson regression coefficient. Statistical analyses were carried out using R software, Version 4.4.1 (http://www.r-project.org/). The level of significance was set at α = .05 (2-tailed) and the false discovery rate (FDR) procedure was used to control false positives over the multiple tests.^21^

## RESULTS

*Participant characteristics:* 235 patients were identified for the study. Of the total patients, there were 104 patients in the methadone group and 119 in the non-exposure group with complete data. The methadone group was significantly younger with a mean age of 44.8 years (SD = 17.1) as compared to 51.1 years (SD = 18.2) in the non-exposure group (*P* = .008). The methadone group had a significantly larger mean %TBSA injury at 12.8% (SD = 11.4) as compared to 7.5% (SD = 7.8) for the non-exposure group (*P* < .001). There were no differences between groups for sex, BMI, or in the percentage of patients with donor site harvest as part of the procedure performed (Table 1).

**Table 1:** Patient Demographics and Injury/Surgery Characteristics

	**Methadone (*n* = 104)**	**Non-exposure (*n* = 119)**	***P*-value (FDR)**
Age, mean (SD)	44.8 (17.1)	51.1 (18.2)	**.008 (0.017)**
BMI, mean (SD)	28.9 (6.17)	28.2 (7.02)	.413 (0.504)
Sex: Male, # (%)	71 (68.3)	86 (72.7)	.613 (0.674)
% TBSA, mean (SD)	12.8 (11.4)	7.37 (7.87)	**<.001 (0.002)**
Donor site, Yes, #(%)	89 (85.6)	93 (78.2)	.210 (0.288)

Boldface script indicates statistically significant *P*-values.

Univariate results unadjusted for the covariates. Total *intraoperative* MME/kg was statistically different between the 2 groups with the methadone group receiving higher mean doses (0.309 vs 0.254, *P* = .013). The methadone group also received more *intraoperative* ketamine on average than the non-exposure group (0.269 mg/kg vs 0.073 mg/kg, *P* < .001). For the 36 h *postoperatively*, the methadone group received higher mean doses of MME/kg as compared to the non-exposure group (0.413 vs 0.383, *P* = .041), as well as more acetaminophen (2770 g vs 1980 g, *P* < .001) and more methocarbamol (1800 g vs 854 g, *P* < .001) (Table 2).

**Table 2:** Univariate Analysis of Medication Administered for Each Respective Group During Study Period

	**Methadone (*n* = 104)**	**Non-exposure (*n* = 119)**	***P*-value (FDR)**
Intraoperative			
MME/kg, mean (SD)	0.309 (0.16)	0.254 (0.16)	**.013 (0.023)**
Ketamine dose/kg, mean (SD)	0.269 (0.33)	0.073 (0.19)	**<.001 (0.002)**
PACU and postoperative period			
MME/kg, mean (SD)	0.413 (0.370)	0.383 (0.240)	**.041(0.504)**
Gabapentin dose (g), mean (SD)	809 (850)	621 (918)	.115 (0.180)
Acetaminophen dose (g), mean (SD)	2770 (2000)	1980 (1590)	**<.001 (0.002)**
Methocarbamol dose (g), mean (SD)	1780 (2080)	850 (1730)	**<.001 (0.002)**

Boldface script indicates statistically significant *P*-values.

Poisson regression adjusted for the covariates. The Poisson regression revealed that, with adjustment for the covariates in Table 3, the administration of methadone decreased opioid consumption by 11% (IRR 0.89, 95% CI: 0.65-1.20, *P* = .447) at 36 h postoperatively, but this effect did not reach statistical significance (Table 3). For the primary outcome of total 36-h postoperative MME/kg, the statistically significant covariates were %TBSA, BMI, and postoperative gabapentin. Clinically, %TBSA was most important, which showed a 2% increase in MME/kg for every 1% increase in TBSA (IRR 1.02, 95% CI [1.01, 1.03], *P* = .003) (Table 3). Additionally, patients in the methadone group had a 9% (IRR 0.91, 95% CI: 0.66-1.24, *P* = .35) reduction in average pain scores in the PACU, and 8% (IRR 0.92, 95% CI: 0.80-1.08, *P* = .31) reduction in average pain scores for the 36 h postoperatively (Table 3). Lastly, patients in the methadone group were discharged home with 14% (IRR 0.86, 95% CI: 0.62-1.19, *P* = .363) fewer opiates (Table 3).

**Table 3:** Poisson Regression for Primary and Secondary Outcomes Variables

**Variable**	**IRR**	**95% CI**	***P*-value**
**Total 36 h postoperative MME/kg**			
Methadone	0.89	(0.65, 1.20)	.447
% TBSA	1.02	(1.01, 1.03)	**.003**
Age	0.99	(0.98, 1.00)	.05
Sex (male)	0.83	(0.60, 1.13)	.222
BMI	0.98	(0.95, 0.998)	**.038**
Donor (Yes)	1.29	(0.89, 1.87)	.179
Intraoperative MME/kg	2.06	(0.85, 5.00)	.105
Intraoperative ketamine mg/kg	0.93	(0.55, 1.57)	.769
Postoperative acetaminophen mg/kg	1.00	(1.00, 1.00)	.787
Postoperative gabapentin mg/kg	1.00	(1.00, 1.00)	**.009**
Postoperative ibuprofen mg/kg	1.00	(0.999, 1.00)	.086
Postoperative methocarbamol mg/kg	0.99	(0.99, 1.00)	.354
**PACU pain scores**			
Methadone	0.91	(0.66, 124)	.35
% TBSA	0.96	(0.94, 0.97)	.53
Age	0.99	(0.98, 1.00)	**<.001**
Sex (male)	1.17	(0.84, 1.64)	.057
BMI	1.02	(0.99, 1.04)	.337
Donor (Yes)	1.13	(0.78, 1.63)	.161
Intraoperative MME/kg	1.67	(0.60, 4.66)	.51
Intraoperative ketamine mg/kg	1.88	(1.12, 3.16)	**.015**
Postoperative acetaminophen mg/kg	1.00	(1.00, 1.00)	.181
Postoperative gabapentin mg/kg	1.00	(1.00, 1.00)	.099
Postoperative ibuprofen mg/kg	1.00	(1.00, 1.00)	.318
Postoperative methocarbamol mg/kg	1.00	(1.00, 1.00)	.072
**Postoperative pain scores**			
Methadone	0.92	(0.80, 1.08)	.31
% TBSA	1.00	(0.997, 1.01)	.231
Age	0.99	(0.989, 0.998)	**.007**
Sex (male)	0.83	(0.70, 0.99)	**.03**
BMI	1.00	(0.99, 1.01)	.981
Donor (Yes)	1.06	(0.87, 1.29)	.534
Intraoperative MME/kg	0.99	(0.58, 1.70)	.973
Intraoperative ketamine mg/kg	0.942	(0.71, 1.26)	.68
Postoperative acetaminophen mg/kg	1.00	(1.00, 1.00)	.356
Postoperative gabapentin mg/kg	1.00	(1.00, 1.00)	.092
Postoperative ibuprofen mg/kg	1.00	(1.00, 1.00)	.391
Postoperative methocarbamol mg/kg	1.00	(1.00, 1.00)	.164
**Discharge MME/kg**			
Methadone	0.86	(0.62, 1.19)	.363
% TBSA	1.01	(0.99, 1.02)	.49
Age	1.01	(1.00, 1.02)	.058
Sex (male)	0.58	(0.42, 0.79)	**<.001**
BMI	0.99	(0.97, 1.02)	.495
Donor (Yes)	0.79	(0.56, 1.11)	.17
Intraoperative MME/kg	26.55	(12.62, 55.83)	**<.001**
Intraoperative ketamine mg/kg	2.91	(1.79, 4.72)	**<.001**
Postoperative acetaminophen mg/kg	1.00	(1.00, 1.00)	**.041**
Postoperative gabapentin mg/kg	1.00	(1.00, 1.00)	.183
Postoperative ibuprofen mg/kg	1.00	(1.00, 1.00)	.508
Postoperative methocarbamol mg/kg	1.00	(1.00, 1.00)	.267

Clinically relevant covariates of age, sex, BMI, %TBSA burned, donor site status, total intraoperative MME/kg, intraoperative ketamine, as well as and postoperative acetaminophen, gabapentin, ibuprofen, and methocarbamol were included in each model to evaluate the independent effect of methadone on the outcomes. Boldface script indicates statistically significant *P*-values.

## DISCUSSION

This is the first study in the adult burn population to describe the effects of intraoperative methadone on pain control and postoperative opioid requirements. The primary aim was achieved. Patients who received a single intraoperative dose of intravenous methadone utilized 11% fewer opioids for 36 h following surgery and demonstrated lower average pain scores in the PACU and for 36 h postoperatively. Additionally, patients who received intraoperative methadone were discharged with 14% fewer opioids. This study identifies an option to reduce the use of postoperative opioids and the associated negative effects in the adult burn population.

Post-anesthesia care unit and postoperative MME/kg decreased with the intraoperative use of methadone and increased with an increase in %TBSA burn. These results are consistent with the pediatric burn literature and the non-burn surgical literature, which show that intraoperative methadone use decreases postoperative opioid requirements. Pain scores were reduced by 9% in the PACU and by 8% for the 36 h postoperatively in the methadone group. Typically, increased opioid administration is associated with improved pain scores. The findings in this study, although not statistically significant, are clinical important, because they suggest an approach in which pain scores can be improved with overall decreased opioid administration.

The results support the existing literature which shows intraoperative methadone can reduce postoperative opioid requirements.^17^ The magnitude of postoperative opioid reduction was smaller in this study and was likely due to the lack of standardized dosing. The range of methadone dose that was administered to our sample patients was 0.05-0.26 mg/kg with a median of 0.166 mg/kg. This is lower than the parenteral dosing cited in the literature, 0.20-0.30 mg/kg.^15^^,^^16^ The lower methadone dose administered in this study may be the result of a flat dosing approach (10 or 20 mg). Although both flat dosing and weight-based dosing are acceptable clinical practices, the literature suggests higher flat doses (20 mg) are needed to reap the benefits of the long elimination half-life of the drug as the pharmacokinetics of smaller doses mimic those of shorter acting opioid.^15^

Gourlay et al. have demonstrated the value of flat dosing of methadone. They administered a 20 mg flat dose of methadone at induction of anesthesia to 23 patients for spine surgery and general surgical procedures, and only 8 study subjects requested opioid medications at 18 h post-surgery.^6^ In a separate study, these same authors administered additional methadone to general surgery and orthopedic patients in the PACU and demonstrated that once these patients were comfortable, no additional opioid was required until 21 h post-surgery.^22^^,^^23^ Gourlay et al. also found a relationship between blood opioid-concentration and pain relief.^22^ In the current study, having blood concentrations of methadone would have been helpful to determine appropriateness of dose administered and may have clarified the diminished effect of the methadone at the 0.05-0.26 mg/kg dosing. We believe that flat dosing contributed to underdosing, which in turn likely contributed to the lack of statistical significance of our results.

Male sex was associated with statistically significant reduction in pain scores and for opioid prescribed at discharge. It is well documented in the literature that differences in sex hormones affect the pain response.^24^ Higher levels of testosterone have been known to increase the pain threshold, while estrogen has been shown to decrease inflammation and pain response.^25^^,^^26^ Jeschke et al. reported a dramatic decrease in beta-estradiol immediately after burn insult while testosterone showed marked decrease four weeks post-burn.^27^ While the females seem to lose the protective effect of estrogen, males still benefit from the improved pain threshold that high testosterone levels allow. This may explain the marked impact on pain perception and opioid need at discharge in the male patients compared to the female patients.

From the Poisson regression, PACU and postoperative opioid use decreased by 7% for each 1 mg/kg of ketamine given and yet its administration was associated with higher pain scores in the PACU. It is common in our institution to co-administer parenteral methadone and ketamine for their synergistic effects during burn debridement. Previous studies in spine surgery have suggested that combining methadone and ketamine results in decreased postoperative narcotic administration.^5^^,^^7^ The mechanism of this synergistic effect is unknown but has been postulated to be due to similar and additive action on NMDA receptors.^8^^,^^28^ Administration of ketamine, while additive, may confound the resulting opioid requirements in the PACU and 36-h post-operative period.

Several factors may have contributed to the higher perception of pain in the PACU of the ketamine exposed group. While not typically seen at analgesic doses, the dissociative effects of ketamine can cause an increase in sympathetic tone and perceived anxiety. These signs and symptoms while emerging from anesthesia may be misunderstood as pain. The physiologic response to burn trauma begins a process of profound inflammation that impacts the central nervous system and pain sensitization.^29–32^ Additionally, the recent literature suggests that inflammatory factors released immediately after burn injury can cause damage to the extracellular matrix of the blood brain barrier.^29^ Leakiness across damaged tight junctions allows entry of inflammatory substances, inducible nitric oxide synthase and nitric oxide. One downstream impact of nitric oxide is the release of increased glutamate. Although there is a potential synergistic effect of methadone and ketamine on NMDA receptor antagonism, the profound inflammatory response and its effects on the central nervous system may confound the picture and have influenced the observed responses in PACU.

The relationship between intraoperative methadone dose and discharge opioid medications is consistent with what we already know about acute and chronic pain syndromes. Subjects who received intraoperative methadone went home with 14% fewer opioids. This may be due to the association described in the literature that early pain control can lead to decreased opioid use later in a hospital stay.^22^ We postulate this decrease may have been greater had the patients received the doses we know to be safe and effective in the literature. Although intraoperative methadone did reduce opioids at discharge, subjects who required higher total intraoperative opiates required more discharge opiates. This may be due to individual patient pain tolerance, pain genetics, preexisting mental health diagnoses (anxiety or depression), or opioid induced hyperalgesia (OIH).^4^^,^^8^ The mechanisms of OIH and opioid-induced tolerance are unclear, but OIH can manifest itself even after very short-term opiate use.^4^^,^^33^

Length of stay was not included in the Poisson regression, which may have influenced duration of prescribed narcotic therapy on discharge. Patients admitted for longer may have consumed opioid for longer duration and been more subject to tolerance and hyperalgesia.^4^ There was also a small subset of patients who were discharged to long term acute care or rehabilitation facilities who were not prescribed narcotics at the end of their hospitalization, but rather at the end of their stay at their post-hospital facility. Their discharge narcotic prescriptions were queried using the Texas Prescription Monitoring Program (PMP). It is important to note that the PMP has historically been used for research purposes. We did not perform specific subgroup analysis regarding these patients, who may have had higher discharge narcotics secondary to longer use of narcotic medicine or other comorbidities affecting narcotic tolerance.

The limitations of this study relate to the retrospective nature, the lack of information on co-morbid conditions and complications, and the subjectivity of pain scores. Specifically, the retrospective nature of the study limited our ability to understand each anesthesiologist’s rationale for their choice of methadone dosing. One possible explanation is that weight-based dose and flat dose administration are both used clinically, and there is a not a single standard practice in anesthesiology. This emphasizes the need for education across our system on methadone dosing, pharmacokinetics, and safety profile.

Information on co-morbid conditions, including obstructive sleep apnea and resultant sensitivity to opioids, pre-injury chronic pain syndromes and prior opioid use, as well as psychiatric diagnoses for which centrally acting medications are used may have provided more insight around the results. Although we did not provide information related to complications from the use of methadone, its safe use in the operating room is well documented.

Finally, reported pain scores remain subjective. The nature of how patients report these scores and how their caretakers use the information for treatment is variable. Pain scores and subsequent opioid administration in the PACU could be confounded by post-operative shivering, physiologic manifestations of hypermetabolic state, or emergence delirium and treatment in the PACU is based on clinical judgement of the healthcare provider.

## CONCLUSION

While the primary results of this retrospective study did not achieve statistical significance, they do highlight important clinical insights and next steps. Intravenous methadone administration in the intraoperative period decreases opioid requirements and pain scores, consistent with literature in other surgical populations. It also has the potential to decrease opioid prescription and use at discharge, an important aspect of long-term pain management for these complex patients who are at high risk for opioid tolerance and hyperalgesia. While the findings reflect outcomes in non-intubated adults with relatively smaller burns (8%-13% TBSA) requiring burn wound excision, there is opportunity to study its use in larger %TBSA burns. There may also be value in studying the use of intravenous methadone for outpatient burn procedures and its impact on opioid use after surgery. The burn community and our patients deserve well-powered, prospective, randomized studies to strengthen the body of literature. Further studies should consider standardized dosing, measured blood drug concentrations and complications associated with methadone use.

## Notes

Author contributions: Christopher R. LaChapelle (Conceptualization [lead], Data curation, Formal analysis [equal], Investigation [lead], Methodology [equal], Project administration [lead], Resources, Software, Supervision, Validation [equal], Writing—original draft, Writing—review & editing [lead]), Aditee Ambardekar (Conceptualization [equal], Data curation [supporting], Formal analysis [equal], Investigation, Methodology [lead], Project administration, Supervision, Writing—original draft, Writing—review & editing [equal]), Jenny Ringqvist (Conceptualization [supporting], Data curation [equal], Formal analysis, Writing—original draft, Writing—review & editing [supporting]), Aiden Berry (Formal analysis [equal], Methodology, Writing—original draft, Writing—review & editing [supporting]), Paul Nakonezny (Formal analysis [lead], Investigation [equal], Methodology, Validation, Writing—original draft, Writing—review & editing [supporting]), Anthony Dao (Data curation [equal], Formal analysis, Writing—original draft, Writing—review & editing [supporting]), and Sarah Rebstock (Conceptualization, Formal analysis, Investigation, Writing—original draft, Writing—review & editing [equal])

Funding: None declared.
