# Correction to: Intraoperative Intravenous Methadone and Postoperative Opioid Requirements in Adult Patients With Burns

**DOI:** 10.1093/jbcr/irag011

**Published:** 2026-01-30

This is a correction to: Christopher R LaChapelle, Aditee Ambardekar, Jenny Ringqvist, Aiden Berry, Paul Nakonezny, Anthony Dao, Sarah Rebstock, Intraoperative Intravenous Methadone and Postoperative Opioid Requirements in Adult Patients With Burns, *Journal of Burn Care & Research*, 2025; iraf209, https://doi.org/10.1093/jbcr/iraf209

The last name of the third author is corrected to read: “Jenny Ringqvist”.

